# The Plight of Healthcare Providers in Managing and Preventing Sexually Transmitted Infections (STIs)/Reproductive Tract Infections (RTIs): A Qualitative Study

**DOI:** 10.7759/cureus.66162

**Published:** 2024-08-05

**Authors:** Divya Madamanchi, Shweta Gangurde, Hetal Rathod, Suman Ray, Akash Nagar, Akhil R, Akhila B S, Gayatri R Nair, Sai Mahesh Vajjala, Shubham Shivale

**Affiliations:** 1 Community Medicine, Dr. D. Y. Patil Medical College Hospital and Research Centre, Dr. D. Y. Patil Vidyapeeth (Deemed to be University), Pune, IND

**Keywords:** dermatologist, gynaecologist, reproductive tract infections, sexually transmitted infections, counsellors, health care providers

## Abstract

Background

Sexually transmitted infections (STIs) and reproductive tract infections (RTIs) pose significant public health challenges globally, particularly in resource-limited settings. This study aimed to investigate the challenges faced by healthcare providers in managing and preventing STIs/RTIs in India.

Materials and methods

In-depth interviews were conducted with eight healthcare providers, including counselors, gynecologists, and dermatologists, working in government settings. A semi-structured interview guide was used to explore challenges related to patient care and healthcare system resources.

Results

The study revealed significant gaps in patient knowledge and awareness about STIs/RTIs, with misconceptions affecting treatment-seeking behavior. Social stigma and cultural barriers were identified as major obstacles to open communication and timely care. Gender-specific challenges in healthcare-seeking behavior and partner notification were noted. Healthcare providers reported inconsistent availability of treatment kits and medications, as well as challenges in ensuring patient compliance. The need for improved healthcare infrastructure, including specialized clinics and better interdepartmental coordination, was highlighted.

Conclusion

Addressing STI/RTI management challenges requires a multifaceted approach, including enhancing public awareness, ensuring consistent medication supply, establishing specialized clinics, and improving interdepartmental coordination. These findings provide valuable insights for developing targeted interventions to improve STI/RTI management and prevention in resource-limited settings.

## Introduction

Sexually transmitted infections (STIs) and reproductive tract infections (RTIs) are formidable public health challenges that significantly impact global health, quality of life, and economic stability. These infections can lead to severe complications such as infertility, various cancers, and pregnancy-related issues. Additionally, they play a crucial role in facilitating the transmission of the human immunodeficiency virus (HIV), thereby exacerbating their impact on both individual health and national economies. The global burden of STIs/RTIs is alarmingly high, with over a million new curable cases occurring daily. In 2012, there were an estimated 357 million new cases of curable STIs among adults aged 15-49 years, including 131 million cases of chlamydia, 78 million cases of gonorrhea, six million cases of syphilis, and 142 million cases of trichomoniasis. Viral STIs also present a significant burden, with around 417 million people infected with the herpes simplex virus type 2 (HSV-2) and approximately 291 million women infected with the human papillomavirus (HPV) at any given time [1].

The emergence of HIV has intensified the focus on STI control due to the strong correlation between the two. In humanitarian settings characterized by prevalent sexual violence and transient job sectors, the risk of STI and HIV transmission is heightened, necessitating comprehensive interventions that address factors like transactional sex and limited access to essential resources such as condoms [2].

The burden of STIs/RTIs is disproportionately heavy in resource-poor regions, with prevalence rates varying widely across different demographics. In India, for example, approximately 6% of the adult population is affected by one or more STI/RTI, resulting in about 30-35 million episodes annually [3]. Across India, reported symptoms and treatment-seeking behaviors vary dramatically, from 64% in Punjab to only 8% in Nagaland. Notably, there has been an increase in the use of public sector healthcare services, rising from 11% in 2005-2006 to 17% currently, while 22% of individuals opt for private sector services. Analysis shows that treatment-seeking is influenced by demographic factors such as age, education, wealth, and employment, with higher odds among women aged 25-35, those with higher education levels, and individuals from wealthier households [4,5].

Studies from different parts of the world reveal that many women do not perceive STIs as personally relevant due to stereotypical beliefs about who is at risk. These beliefs negatively impact reactions to diagnosis, causing anxiety about disclosure, especially to sexual partners, and concerns about future reproductive health [6]. Women often experience fear of stigma and self-blame for contracting STIs like chlamydia. In contrast, men tend to show less concern, hesitate to disclose their condition to their partners, and sometimes blame their partners. Delays in seeking care are often linked to the perception of STIs, particularly chlamydia, as minor infections, especially among men [7]. Higher rates of stigma and shame are reported among those who have not undergone gonorrhea or HIV testing in the past year. Factors influencing gonorrhea testing include sex, age, health service utilization, previous gonorrhea suspicion, and low stigma levels. For HIV testing, influential factors include age, enrolment site, health service use, gonorrhea testing, and low stigma levels. Among females, stigma (but not shame) is associated with anticipating negative reactions to disclosing sexual behaviors to healthcare providers (HCPs) and seeking STD-related care in the past year, while no such association is found among males. The perceived seriousness of symptoms plays a role in influencing STD-related care-seeking behavior, with cognitive and emotional components potentially acting independently [8,9].

In response to these challenges, the National AIDS Control Programme Phase V (NACP V) has emphasized the importance of engaging both the public and private sectors in providing STI/RTI services. Adherence to national guidelines is crucial for standardizing and improving STI/RTI management and mitigating the risk of drug resistance. Despite these efforts, treatment regimens differ significantly between the public and private sectors. While the National AIDS Control Organisation (NACO) advocates for a syndromic approach, private HCPs often rely on clinical judgment for STI/RTI management [10].

A study conducted in Vadodara, India, aimed to understand HCPs' perspectives on STI/RTI management. It revealed that herpes genitalis was the most common STI reported by 75% of skin and VD specialists but only 2.27% of general practitioners (GPs). Obstetricians and gynecologists identified *Trichomonas vaginalis* as the most common STI in 32.35% of cases, whereas 43% of GPs reported gonorrhea as the predominant STI/RTI condition in their practice. The study also highlighted that some patients seek treatment from non-allopathic HCPs or indigenous healers, possibly due to misconceptions or a lack of awareness about proper STI/RTI management. Furthermore, female patients often prefer consulting obstetricians and gynecologists, especially female doctors, for STI/RTI conditions [11].

Another qualitative study by Chesang K. et al. focusing on HCPs' perspectives on managing STIs in HIV settings in Kenya emphasized the challenges faced by HCPs. Despite recognizing the high priority of STI management, HCPs were hampered by inadequate supervision, insufficient reporting, and discontinued STI training programs. Poor prevention strategies, particularly regarding medication compliance and contact tracing, contributed to the rising STI cases and associated stigma. HCPs frequently encountered STIs in the population but faced barriers such as frequent medication stock-outs, neglect of specific STIs like anal infections, and a perceived low priority for STI management. The study also noted selection biases and regional exclusions, potentially affecting the comprehensiveness of the findings [12].

Addressing the global burden of STIs/RTIs requires a multifaceted approach, including robust health education at the community, patient, and HCP levels, ensuring access to treatment in a supportive and nonjudgmental atmosphere, and standardizing treatment regimens across all sectors. Effective management strategies and reducing the stigma associated with STIs are critical to improving public health outcomes and mitigating the broader social and economic impacts of these infections. Our study aims to understand the challenges faced by HCPs in the management and prevention of STIs/RTIs.

## Materials and methods

Data collection

In depth interviews with HCPs (and counsellors) involved in the management of STIs/RTIs were taken to evaluate the problems faced by the practitioners in terms of management and prevention of STIs/RTIs. Purposive sampling was used, and an interview guide (Annexure) was developed to gather relevant information.

In order to gain a better understanding of the topic, HCPs were interviewed face-to-face using in-depth interviews in a separate room at their respective work facility. The aim of the study was explained to participants before starting the interview. Participants who fulfilled the inclusion criteria were taken in the study, and informed consent for the audio recording was taken before starting the interview. Throughout the interview, notes were taken to capture both verbal and non-verbal data.

A total of eight interviews were conducted, which included one STI/RTI counselor, four dermatologists, and three gynecologists. The interviews typically lasted between 10 and 23 minutes.

Study tools

An interview guide was made to assess the “Challenges Faced by HCPs in Prevention and Management of Sexually Transmitted Infections (STIs) and Reproductive Tract Infections (RTIs).”

The interview guide focused on challenges faced with respect to the patients, drug supply, health programs, and other resources.

Inclusion criteria

HCPs (STI/RTI counselors, dermatologists, and gynecologists) from government settings who were willing to give interviews were selected.

Exclusion criteria

HCPs working in private settings and those working in government settings but not giving consent were excluded.

## Results

Theme 1: clinical presentation of the patients

This theme covers the demographic characteristics of patients presenting with STIs and RTIs such as their age, gender, and socioeconomic status. It also includes the common clinical symptoms and presentations observed in these patients.

Subtheme 1.1: Common Symptoms and Gender Differences

This subtheme highlights the typical symptoms reported by male and female patients, with women commonly presenting with vaginal discharge and men with genital ulcerations and urethral discharge.

"We generally see female patients presenting with vaginal discharge. And in male patients, genital ulcerations are most common." (Interview 3)

"Commonly we see patients with urethral discharge or urethral ulcers. Female patients also we do see from time to time, but they are much fewer than the male patients and female patients will come with vaginal discharge." (Interview 7)

"Mostly, vaginal discharge. Chronic PV discharge, continuous dirty, white discharge." (Interview 2)

"Vaginal discharge and lower abdomen pain are commonly seen. We give them kit no. 2 for vaginal discharge and kit no. 6 for lower abdomen pain." (Interview 1)

"Male patients are syphilis positive. They come once or twice a month." (Interview 1)

Subtheme 1.2: Age Distribution

This subtheme focuses on the age range in which STIs and RTIs are most prevalent among the patients visiting the clinics.

"In our OPD, STIs are most common between 20 to 40 years of age." (Interview 3)

"Mostly from 20 to 40 years of age." (Interview 4)

"We see about 2-4 cases of vaginal discharge in patients below 20 years." (Interview 4)

"More than 30 years old patients are usually seen." (Interview 1)

Theme 2: patient knowledge, awareness, and misconceptions

This theme delves into the level of knowledge and awareness that patients have about STIs and RTIs, including common misconceptions that can impact their treatment-seeking behavior and compliance.

Subtheme 2.1: Limited Awareness and Knowledge About STIs/RTIs

This subtheme highlights the general lack of awareness and knowledge among patients regarding STIs and RTIs, their transmission, and their potential consequences.

"Patients generally do not have much knowledge. They might know that HIV is a sexually transmitted disease, but they are unaware of other STDs and the various ways these diseases can spread." (Interview 5)

"So, the patients who presented to us, yes they were not aware about STIs, RTIs." (Interview 7)

"Many don't openly talk about their symptoms initially, but after taking a history, we find out more." (Interview 2)

"Many don't realize that their condition is sexually transmitted. They are generally unaware of the transmission methods." (Interview 5)

"They think it's only through vaginal intercourse. They don't consider oral sex, blood products, or needle pricks as possible ways of transmission." (Interview 6)

Subtheme 2.2: Misconceptions About Transmission and Treatment

This subtheme focuses on the various misconceptions held by patients, such as believing that they cannot contract an STI if their partner does not exhibit visible symptoms or that treatment is unnecessary in certain cases.

"The most common misconception is that if their partner doesn't have lesions, they believe they don't have an STI." (Interview 6)

"Many think treatment isn't necessary, especially for conditions like syphilis without visible lesions." (Interview 3)

"People do believe that they are in denial that it may be an STI. They believe that it could be something else; some people even believe it could just be a fungal infection." (Interview 7)

"Yes, there are misconceptions and cultural barriers that prevent people from openly talking about sexual health." (Interview 2)

Subtheme 2.3: Impact on Treatment-Seeking Behavior

This subtheme explores how the lack of awareness and misconceptions can negatively impact patients' willingness to seek timely and appropriate treatment, potentially leading to complications or further transmission of infections.

"Yes, definitely. Many patients are unaware that they have conditions like syphilis until it affects their pregnancy, which can harm the fetus." (Interview 5)

"Yes, the stigma does hamper the treatment-seeking behavior." (Interview 7)

"Yes, lack of awareness. TV ads are not informative enough." (Interview 2)

"It's also challenging because partners often do not accompany the patients to the clinic, making it difficult to get a complete history." (Interview 5)

Theme 3: patient attitudes, emotions, and stigma

This theme examines the emotional responses and attitudes of patients when diagnosed with or seeking treatment for STIs and RTIs, as well as the social stigma associated with these conditions.

Subtheme 3.1: Fear, Anxiety, and Embarrassment

This subtheme explores the feelings of fear, anxiety, and embarrassment that patients often experience when confronted with an STI or RTI diagnosis, which can impact their willingness to disclose information or seek proper treatment.

"They are scared." (Interview 1)

"They get frightened." (Interview 1)

"Initially, they do not realize it's a sexually transmitted disease. Once they understand, they may feel shy or guilty." (Interview 4)

"There is definitely a stigma, they feel like it is a stigma and hence they will try to give an alternate history instead of the correct one." (Interview 7)

"Yes, especially younger patients or those from LGBTQ+ communities fear societal judgment." (Interview 5)

Subtheme 3.2: Reluctance to Disclose Symptoms and Seek Treatment

This subtheme highlights the reluctance of patients to openly discuss their symptoms or seek medical attention due to fear, embarrassment, or a lack of understanding about the severity of their condition.

"Many don't openly talk about their symptoms initially, but after taking a history, we find out more." (Interview 2)

"They are quite anxious. I believe a lot of them will also try to hide a history of exposure." (Interview 7)

"Yes, they are usually reluctant to speak about their complaints. Once they open up, they often have limited knowledge about their partner's condition and might not be aware of their partner's symptoms." (Interview 5)

"Sometimes it happens that patients do not even tell that they have had sexual exposure, but then after giving 20 minutes, 25 minutes talking to them, they suddenly tell." (Interview 4)

Subtheme 3.3: Social Stigma and Cultural Barriers

This subtheme addresses the social stigma and cultural barriers that can prevent patients from discussing or seeking treatment for STIs and RTIs, as these conditions are often viewed as taboo or shameful in certain societal contexts.

"People feel shy." (Interview 1)

"Yes, there are misconceptions and cultural barriers that prevent people from openly talking about sexual health." (Interview 2)

"Females don't talk openly to either male or female doctors, especially if accompanied by their spouse." (Interview 3)

"Some people go to Ayurveda." (Interview 1)

"Yes, about 30% have received incomplete treatment or seen unqualified providers." (Interview 5)

Theme 4: treatment and compliance

This theme focuses on the various aspects of treating STIs and RTIs, including treatment protocols, medication availability, patient compliance, and the use of alternative or self-medication practices. Effective treatment is crucial for managing these infections, preventing complications, and mitigating further transmission.

Subtheme 4.1: Treatment Protocols and Medication Availability

This subtheme outlines the standard treatment protocols and medications used for managing STIs and RTIs while also highlighting potential issues with the availability of certain medications or treatment kits.

"For vaginal discharge, we use a green kit with fluconazole and secnidazole. For genital ulcers, a white kit with benzathine penicillin and azithromycin." (Interview 3)

"Mostly the green kit." (Interview 6)

"If the kit is unavailable and the patient is willing, we prescribe the required medications." (Interview 6)

"Yes, there are issues with the availability of the syndromic approach kits (yellow and green kits)." (Interview 2)

"We usually have the necessary kits, though sometimes there are shortages of specific kits." (Interview 4)

Subtheme 4.2: Patient Compliance and Follow-Up

This subtheme examines the level of patient compliance with prescribed treatment regimens and the importance of follow-up appointments for the effective management of STIs and RTIs. It also explores the factors that contribute to non-compliance.

"It's about 50-50. Some are compliant, but many take only a few pills and don't complete the course." (Interview 2)

"I would say maybe 90-95% of them do follow-up because then they do understand that if they don't follow-up that it could become a very dangerous disease." (Interview 7)

"The more time you give to counseling, the more they are willing to follow up." (Interview 6)

"We counsel them and follow up through the ART center, which calls them to remind them to resume treatment." (Interview 6)

"I'd say about 50% comply with the treatment. Some people think taking a few pills is enough." (Interview 2)

Subtheme 4.3: Use of Alternative and Self-Medication Practices

This subtheme discusses the prevalence of alternative and self-medication practices among patients, including the use of traditional or non-prescribed treatments, and the potential risks associated with these practices.

"Yes, many patients seek treatment from general practitioners, take antibiotics for other infections, and then present to us with STIs." (Interview 2)

"Yes, some use traditional medicine and eventually come to us when it doesn’t help." (Interview 4)

"Yes, they take over-the-counter medications or consult unqualified practitioners." (Interview 6)

"Some patients seek help from alternative medicine practitioners, which delays proper treatment." (Interview 5)

Theme 5: barriers to effective treatment

This theme focuses on the challenges and barriers that hinder the effective treatment and management of STIs and RTIs, including issues related to healthcare access, medication availability, and patient education. Addressing these barriers is crucial for improving patient outcomes and public health.

Subtheme 5.1: Healthcare Access and Systemic Challenges

This subtheme explores the various challenges related to accessing healthcare services, such as long waiting times, lack of specialized clinics, and issues with referral systems, which can impact the timely diagnosis and treatment of STIs and RTIs.

"Yes, patients often seek treatment at PHCs where proper referral systems are lacking." (Interview 6)

"Patients usually seek treatment from PHCs and CHCs before reaching us." (Interview 4)

"Patients do not go to government hospitals because they think private doctors are better." (Interview 2)

"Yes, patients seek initial treatment from PHCs before being referred to us." (Interview 5)

"We receive patients referred from PHCs, who are then given the prescribed kits and follow-ups." (Interview 4)

Subtheme 5.2: Education and Awareness Programs

This subtheme emphasizes the importance of education and awareness programs in improving patient knowledge about STIs and RTIs, promoting early diagnosis and treatment, and addressing misconceptions that hinder effective management.

"We hold awareness programs on specific days like World AIDS Day." (Interview 2)

"Education on STI and HIV prevention is essential for improving patient outcomes." (Interview 3)

"Patients are generally unaware, and there's a need for more targeted education campaigns." (Interview 5)

"Yes, awareness is low, and there's a need for continuous education to address this." (Interview 6)

Subtheme 5.3: Cultural and Social Barriers

This subtheme discusses the cultural and social barriers that prevent patients from seeking treatment, such as stigma, discrimination, and the reluctance to discuss sexual health issues openly, and suggests ways to address these barriers.

"People feel shy about discussing STIs." (Interview 1)

"Yes, cultural barriers and stigma prevent open discussions about sexual health." (Interview 2)

"Females are hesitant to talk about their symptoms, especially if accompanied by their spouse." (Interview 3)

"There is a stigma associated with STIs, which makes patients reluctant to seek help." (Interview 7)

"Some patients prefer traditional or alternative medicine due to cultural beliefs." (Interview 6)

Theme 6: training and support for HCPs

This theme addresses the training, support, and resources available to HCPs for the diagnosis, treatment, and management of STIs and RTIs, including the need for ongoing education and professional development.

Subtheme 6.1: Training Programs and Guidelines

This subtheme highlights the importance of regular training programs and updated clinical guidelines for HCPs to ensure they are well-equipped to diagnose and treat STIs and RTIs effectively.

"Yes, we receive training on updated protocols and treatment guidelines." (Interview 6)

"We have regular training sessions and workshops on STI management." (Interview 5)

"Yes, the staff receives periodic training on new guidelines and treatment protocols." (Interview 4)

"We have guidelines provided by the government, which we follow strictly." (Interview 2)

"Training programs are essential for keeping us updated with the latest treatment protocols." (Interview 3)

Subtheme 6.2: Support Systems and Resources

This subtheme explores the availability and effectiveness of support systems and resources, such as counseling services, diagnostic tools, and patient education materials, that assist HCPs in managing STIs and RTIs.

"We have access to counseling services and educational materials for patients." (Interview 6)

"Support from other healthcare professionals and referral systems is crucial for comprehensive patient care." (Interview 5)

"We have diagnostic tools and kits available, although there are occasional shortages." (Interview 4)

"Patient education materials and counseling services are available to support our work." (Interview 2)

"Yes, we have support systems in place, including counseling services and patient education programs." (Interview 3)

Subtheme 6.3: Need for Ongoing Professional Development

This subtheme emphasizes the need for continuous professional development and education so that HCPs can stay updated with the latest advancements in STI and RTI management and improve patient care outcomes.

"Yes, ongoing professional development is essential for staying updated." (Interview 6)

"Continuous education and training are necessary for effective STI management." (Interview 5)

"We need regular updates and professional development opportunities to enhance our skills." (Interview 4)

"Ongoing professional development helps us stay informed about new treatment options and guidelines." (Interview 2)

"Yes, continuous learning and professional development are crucial for improving patient care." (Interview 3)

## Discussion

Clinical presentation of the patients

The analysis reveals important patterns in the clinical presentation of STIs and RTIs. There are notable gender differences in symptom presentation, with women more commonly reporting vaginal discharge and lower abdominal pain, while men frequently present with genital ulcerations and urethral discharge. This gender-specific symptomatology is crucial for HCPs to recognize, as it can guide initial diagnostic approaches and treatment decisions.

The age distribution of patients seeking treatment for STIs and RTIs is predominantly between 20 and 40 years old, which aligns with the period of peak sexual activity in most populations. However, there are also reports of cases in patients under 20 years old, highlighting the need for sexual health education and services tailored to adolescents and young adults. The prevalence of STIs in this age group underscores the importance of early intervention and prevention strategies targeted at youth.

These findings emphasize the need for HCPs to be vigilant about age- and gender-specific presentations of STIs and RTIs. They also suggest that public health initiatives should focus on educating both men and women about the various symptoms associated with these infections to promote early recognition and treatment-seeking behavior. HCPs also mentioned men who have sex with men (MSMs) visiting the clinics on a regular basis, with one practitioner mentioning the ratio of MSMs to other patients to be 1:20.

A study in Vadodara, India, examined HCPs' perspectives on STI/RTI management and found that herpes genitalis was reported as the most common STI by 75% of skin and VD specialists and 2.27% of GPs. For obstetricians and gynecologists, 32.35% reported *Trichomonas vaginalis *as the most common STI in their practice, and 43% of GPs reported gonorrhea as the most common STI/RTI condition in their practice [11].

Patient knowledge, awareness, and misconceptions

The analysis reveals a concerning lack of awareness and knowledge about STIs and RTIs among patients. Many individuals are unaware of the various types of STIs beyond HIV, and there is a significant gap in understanding regarding transmission methods. This lack of knowledge can lead to risky behaviors and delayed treatment-seeking, potentially exacerbating the spread of infections.

Common misconceptions identified include the belief that STIs cannot be transmitted if a partner shows no visible symptoms and that treatment is unnecessary for asymptomatic conditions like syphilis. These misconceptions can have serious consequences, particularly in cases where asymptomatic infections can lead to complications or transmission to partners or fetuses during pregnancy.

The impact of this knowledge deficit on treatment-seeking behavior is significant. Many patients delay seeking care due to a lack of awareness about the potential seriousness of their symptoms or the availability of effective treatments. Cultural barriers and stigma further compound this issue, preventing open discussions about sexual health and creating additional obstacles to seeking timely medical attention.

To address these challenges, there is a clear need for comprehensive sexual health education programs that go beyond basic HIV awareness. These programs should address common misconceptions, emphasize the importance of regular testing and early treatment, and provide accurate information about the various types of STIs and RTIs, their symptoms, and transmission methods. Additionally, efforts to reduce stigma and promote open dialogue about sexual health could help overcome cultural barriers to seeking care. Similar studies mentioned patients’ lack of awareness and significant gaps in communication skills among HCPs, along with discomfort discussing sexual health and the stigma surrounding STIs, hindered effective patient engagement and disclosure [11,12].

Patient attitudes, emotions, and stigma

The analysis highlights the significant emotional and psychological impact of STI/RTI diagnoses on patients. Fear, anxiety, and embarrassment are common reactions, often rooted in the societal stigma surrounding these infections. These emotional responses can profoundly influence patients' behavior, leading to reluctance to disclose symptoms, seek treatment, or inform partners.

The reluctance to openly discuss symptoms or seek medical attention is a critical barrier to effective STI/RTI management. Patients often attempt to hide their history of sexual exposure or provide alternative explanations for their symptoms, complicating diagnosis and treatment. This behavior is particularly pronounced among younger patients and those from LGBTQ+ communities, who may fear additional judgment or discrimination.

Social stigma and cultural barriers play a significant role in shaping patients' attitudes and behaviors. In many societal contexts, STIs and RTIs are viewed as taboo or shameful, leading to secrecy and avoidance of necessary medical care. This stigma can also result in patients seeking treatment from unqualified providers or resorting to ineffective alternative treatments, potentially worsening their condition and increasing the risk of transmission.

To address these issues, HCPs and public health initiatives should focus on creating non-judgmental, confidential environments for STI/RTI screening and treatment. Empathetic counseling and education can help alleviate patients' fears and encourage more open communication. Additionally, broader societal efforts to destigmatize sexual health issues could contribute to more positive attitudes and health-seeking behaviors.

A qualitative study done by Chesang K et al. in Kenya showed that poor prevention approaches, particularly in medication compliance and contact tracing, contributed to rising STI cases and stigma [12].

Treatment approaches and patient compliance

The analysis reveals both standardized approaches to STI/RTI treatment and challenges in their implementation. Treatment protocols typically involve the use of specific medication kits for different syndromes such as vaginal discharge or genital ulcers. However, the availability of these kits can be inconsistent, forcing HCPs to adapt by prescribing alternative medications when necessary.

Patient compliance with prescribed treatments emerges as a significant concern. Compliance rates vary widely, with some HCPs reporting that only about 50-60% of patients complete their full course of treatment. Factors contributing to non-compliance include a lack of understanding about the importance of completing treatment, financial constraints, and the stigma associated with repeatedly visiting clinics for STI/RTI care.

The practice of self-medication and seeking alternative treatments from unqualified practitioners is another challenge highlighted in the analysis. Many patients attempt to treat themselves with over-the-counter medications or consult "quacks" before seeking proper medical care. This can lead to delayed or ineffective treatment, potentially allowing infections to progress or spread. Other similar studies mention that some patients seek treatment from non-allopathic HCPs or indigenous healers, which could be related to misconceptions or lack of awareness about proper STI/RTI management [11]. Another study mentioned that frequent shortages of essential STI medications forced HCPs to prescribe alternatives outside treatment guidelines, compromising treatment effectiveness and increasing the risk of drug resistance [12]. A major limitation found in the study is that few medical officers and counselors, when approached, declined to participate in the research. This unforeseen challenge not only affected the study's sample size but also raised questions about the broader attitudes toward STI/RTI research among healthcare professionals.

## Conclusions

STIs and RTIs pose significant challenges in healthcare. Patients often lack awareness and hold misconceptions. Fear and stigma hinder treatment-seeking. Symptoms vary by gender, with women reporting vaginal discharge and men presenting genital ulcers. The 20-40 age group is most affected. Treatment protocols exist but face implementation issues. Medication availability is inconsistent. Patient compliance is low, around 50-60%. Self-medication complicates management. Healthcare systems struggle with limited specialized clinics and resources. Interdepartmental coordination needs improvement. The MSM community faces unique challenges. Addressing these issues requires comprehensive strategies. These include public education, stigma reduction, improved healthcare infrastructure, and tailored interventions. Enhancing awareness, ensuring resource availability, and promoting open communication are crucial. With concerted efforts, STI/RTI management and prevention can be significantly improved.
